# Alterations in Mitochondrial and Endoplasmic Reticulum Signaling by p53 Mutants

**DOI:** 10.3389/fonc.2016.00042

**Published:** 2016-02-25

**Authors:** Carlotta Giorgi, Massimo Bonora, Sonia Missiroli, Claudia Morganti, Giampaolo Morciano, Mariusz R. Wieckowski, Paolo Pinton

**Affiliations:** ^1^Section of Pathology, Oncology and Experimental Biology, Laboratory for Technologies of Advanced Therapies (LTTA), Department of Morphology, Surgery and Experimental Medicine, University of Ferrara, Ferrara, Italy; ^2^Department of Biochemistry, Nencki Institute of Experimental Biology, Warsaw, Poland

**Keywords:** calcium, mitochondria, endoplasmic reticulum, apoptosis, cell death

## Abstract

The p53 protein is probably the most important tumor suppressor, acting as a nuclear transcription factor primarily through the modulation of cell death. However, currently, it is well accepted that p53 can also exert important transcription-independent pro-cell death actions. Indeed, cytosolic localization of endogenous wild-type or transactivation-deficient p53 is necessary and sufficient for the induction of apoptosis and autophagy. Here, we present the extra-nuclear activities of p53 associated with the mitochondria and the endoplasmic reticulum, highlighting the activities of the p53 mutants on these compartments. These two intracellular organelles play crucial roles in the regulation of cell death, and it is now well established that they also represent sites where p53 can accumulate.

## Introduction

The p53 protein is the product of one of the most frequently mutated tumor suppressor genes in human cancer (TP53), playing a crucial role in the response of a myriad of intracellular pathways (1). Despite this master role, more than 50% of human cancers harbor somatic p53 gene mutations (2).

Unlike most tumor suppressor genes, which are predominantly inactivated by deletions or truncating mutations during cancer progression, the TP53 gene in human tumors often contains missense mutations that produce a full-length protein containing only a single amino acid substitution (called naturally occurring mutants) with a greatly prolonged half-life (3, 4).

Mutations in p53 result in both loss-of-function and gain-of-function activities (5–7).

In addition to its nuclear activity, there is much evidence to support the idea that the cytoplasmic pool of p53 plays a pivotal role in inducing apoptosis through a transactivation-independent mechanism (8) and Table 1.

**Table 1:** **p53 mutants localization and function**.

P53 mutant	Localization	Ability to induce apoptosis	Transcription independent	Reference
L-p53	M	Yes	Yes	(9)
R273H	C	No		(10)
R175H	N, C	No		(11)
L194F	N, C	No		(10)
R280K	N, C	No		(10)
G245S	N, C	No		
R248W	N, C	No		(11)
R249S	N, C	No		(11)
R282W	N	No		(11)
L22Q	N	Yes	Yes	(12)
W23S	N	Yes	Yes	(12)
ΔNLS	C	Yes	Yes	(13)
dl214	N, C	Yes	Yes	(14)
N1–102	C	Yes	Yes	(13)
M246I	N	No		(15)

*N, nucleus; M, mitochondria; C, cytosol*.

The overexpression of a mutant p53, lacking most of the DNA-binding domain (DBD) and completely deficient in transactivation function, still triggers apoptosis (14). Moreover, the overexpression of a number of transactivation-incompetent p53 mutants efficiently induces apoptosis (16). Similarly, it was shown that p53 is able to trigger apoptosis even in the absence of a nucleus (13). However, these studies did not provide any mechanistic explanation; only several years later, a breakthrough study showed a direct role of p53 in mitochondrial apoptosis (17).

Mitochondria play a pivotal role in cell death (18), including apoptosis. In healthy cells, the inner mitochondrial membrane, the barrier between the intermembrane space (IMS) and the matrix, is nearly impermeable to all ions. Mitochondrial membrane permeabilization, e.g., through opening of the permeability transition pore (PTP) (19), is frequently the decisive event that delineates the balance between survival and death (20, 21).

Proapoptotic signals resulting in outer mitochondrial membrane (OMM) permeabilization induce the release of IMS proteins that, once in the cytosol, activate different cell death-associated pathways (22).

How p53, once at the mitochondrion, induces OMM permeabilization to trigger the release of proapoptotic factors from the IMS is described in depth in the next section.

The endoplasmic reticulum (ER) can connect to and act synergistically with other membranous structures, including mitochondria, in particular at ER–mitochondria contact sites, also known as mitochondria-associated ER membranes (MAMs), which have a distance of approximately 10–25 nm between the ER and the mitochondria (23, 24). The close proximity of the ER and the OMM explains how proteins situated on the opposing membrane faces could interact and thus “tether” the two organelles (25, 26).

The ER and the mitochondria reciprocally transmit danger signals through physical contacts (24, 27). In particular, the ER–mitochondrial cross talk plays a key role in decoding Ca^2+^-mediated apoptotic signals (28–32).

## p53 and the Mitochondria

Moll and colleagues showed that death signals induce p53 stabilization and rapid translocation (30–60 min) to the mitochondria in primary, immortal, and transformed cells (9, 17, 33). The main evidence for a sequence-specific transactivation (SST)-independent pathway for p53-induced apoptosis comes from Mihara et al., who demonstrated that bypassing the nucleus by targeting p53 to the mitochondria (*via* fusion with a mitochondrial import leader peptide, designated L-p53) was sufficient to launch apoptosis in p53-deficient tumor cells (9) and to suppress colony growth, although not as strongly as nuclear p53.

When a portion of endogenous p53 trafficked to the mitochondria, it interacted with the Bcl-2 family members, including the anti-apoptotic Bcl-2 and Bcl-xL at the OMM to block their functions (9). Interestingly, through nuclear magnetic resonance (NMR) spectroscopy, it has been demonstrated that the p53 DBD is involved in this protein–protein interaction (34). Indeed, mutant p53-harboring breast cancer cells, such as MDA 468, SKBr3, T47D, and MDA 231 (missense p53 mutations R273H, R175H, L194F, and R280K, respectively, both structural and DNA-binding mutant types), were unable to elicit this interaction (34).

DNA-binding domain represents the central core domain of p53 structure required for sequence-specific DNA binding (residues 102–292), and it is the most highly conserved region where more than 80% of p53 mutations occur.

**Figure 1:**
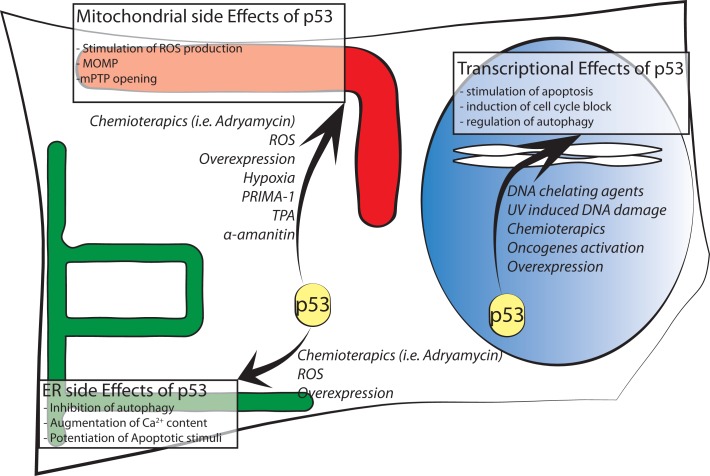
**Schematic representation of p53 intracellular localizations known to date**. Major effects of intracellular distribution are shown in boxes while agents stimulating p53 localization are in italic font.

Moreover, trafficking wild-type (wt) p53 within the mitochondria displayed the activation of proapoptotic members, such as Bax and Bak (13, 35, 36), to induce their oligomerization by forming a pore in the OMM. As a result, the oligomerization allowed the release of cytochrome *c* into the cytoplasm, resulting in apoptosis induction (33). To gain functional insight, they were brought forward *in vitro* experiments using purified recombinant p53 proteins at increasing concentrations. Because modifications in the p53 DBD (represented by the p53 R175H and p53 R273H naturally occurring mutants) were strictly unable to promote cytochrome *c* release and the fact that p53 L194F, R280K, G245S, R248W, R249S, and R282W missense mutants destroyed any attempt to mediate OMM permeabilization (Figure 1), the integrity of the p53 DBD was declared essential for SST-independent apoptosis as well as for transcription-independent cell death.

In contrast, conflicting results can be seen in previously published papers, in which specific mutations that abolish the trans-activating functions of p53 (L22Q and W23S) in the amino-terminal domain (residues 1–42) or impair the p53 nuclear localization signal (p53 ΔNLS) in the C-terminal region (residues 324–393) did not fully abolish the p53 apoptogenic potential (14).

In 1995, Haupt and coworkers reported that a truncated p53 protein, containing only the first 214 residues of wt p53 (p53 dl214) and incapable of SST, appeared as a potent inducer of apoptosis. Similar experiments were conducted with a protein containing two point mutations (p53 Gln22Ser23), and in this case, the ability to induce efficient apoptotic cell death without SST properties, even though it occurred more slowly, was also confirmed (14). All of these sharply contradictory results culminated in the discovery of a p53 N1–102 truncation mutant as the minimal requirement for proapoptotic activity. This protein includes a mutated transactivation domain and an intact proline-rich region (residues 61–94 containing multiple copies of the PXXP sequence, where X is an amino acid) but lacks the central core and the C-ter previously mentioned. On the basis of these findings, p53 death signaling seems to depend on the proline-rich regulatory domain and does not require transactivation of target genes (13). Thus, as consequence, the DBD may be dispensable. The proline-rich region plays a role in p53 stability regulated by mouse double minute 2 homolog (MDM2). Indeed, p53 becomes more susceptible to degradation by MDM2 if this region is deleted (37).

To be thorough, it should be considered that p53 mutants bind their interactors also in a DNA structure-selective mode as well as in a sequence-specific manner (38). However, this topic needs to be explored by additional studies.

Recently, it has been demonstrated how wt p53 can contribute to the apoptotic process through the caspase 3 protein. First, Sayan et al. demonstrated the presence of two caspase 3 cleavage sites in wt p53 at residues Asp^21^ and Asp^186^. Because a portion of both proteins localized to the mitochondria, these authors compared the apoptotic activities of wt p53, non-cleavable mutants (D21A and D186A), and naturally occurring p53 mutants (R248W and R249S) (39). Their results, through overexpression experiments, suggested that following caspase activation p53 gains a transcription-independent function to reinforce apoptosis, leading to the formation of a positive feedback loop in which p53 accumulation induces caspase cleavage and promotes apoptosis. Later, Frank and coworkers identified and characterized pro-caspase-3 as a mitochondrial p53-interacting protein. This finding was detectable in wt p53 cells, upon stress induction and following Adriamycin treatment, but only 1–3% of the total wt p53 had this affinity. p53 R175H and R273H stably transfected cells shown how both the mutants were able to interact with caspase-3 at levels comparable to wt p53, but conversely impaired the ability of pro-caspase-3 to be activated by upstream caspases (40).

More recently, Sorrentino showed the strong requirement of the phosphorylation-specific peptidyl-prolyl cis/trans isomerase, NIMA-interacting 1 (Pin1) in the early phases of p53-dependent apoptosis by controlling its mitochondrial accumulation, activation, and increased retention (41). Phosphorylation at Ser46 on p53 by the homeodomain-interacting protein kinase 2 (HIPK2) is a key event in promoting its monoubiquitination and translocation to the mitochondria. These findings were intimately interconnected with the results previously obtained by Mihara and Mancini regarding the p53–Bcl-2 interaction and Ser46 phosphorylation of p53 (9, 42), and those results reported by Dumont et al. on the impact of the p53 codon 72 Pro/Arg polymorphism on the direct mitochondrial activity of p53 (37). In particular, the Arg72 displayed a higher level of translocation to the mitochondria, with this variant found to have higher phosphorylation on Ser46 and to be bound to Pin1 better than the Pro72 counterpart.

A small molecule known as PRIMA-1, which activates p53 to achieve tumor suppression by restoring the capacity of mutant protein to bind DNA, has been shown to induce the death of tumor cells expressing p53 and tumor-derived mutation p53 M246I and p53 R273H in the absence of transcription (13, 43).

Finally, another functional link between p53 and mitochondria originates from reactive oxygen species (ROS). ROS may contribute as actors on the stage of mitochondria–nuclear communication, interfering with the activity of various protein kinases and phosphatases (44, 45). Changes in mitochondrial function involving alterations in ROS production could affect p53 activity and its subcellular localization. In one specific case, p53 underwent an amino acid redox modification at cysteine 277 (oxidation of Cys277) in the p53 DBD that altered its DNA-binding affinity (46). Moreover, mitochondria-generated ROS induce p53 translocation to the mitochondria and in turn stimulate oxidative stress (46, 47) in part by transcription-independent mechanisms, leading to an increase in apoptosis. p53 translocates not only to the OMM but also into the matrix, where it was able to interact with protein–manganese superoxide dismutase (MnSOD, Figure 1), leading to a reduction of its scavenging activity [Ref. (48) or Ref. (49) for an extensive review].

In the mitochondrial compartment, p53 has also been shown to accumulate following oxidative stress and ischemia, where it triggered PTP opening and necrosis (50). Thus, these results reveal a new role for p53 in activating necrotic cell death (Figure 1).

In the mitochondrial matrix also resides p53Ψ, an isoform product of an alternative 3′ splice-site activation of p53 mRNA. Consistent with previous information, this protein lacks SST functions and is sufficient to promote prometastatic features in epithelial cells (51). Moreover, cells expressing p53Ψ exhibited increased mitochondrial permeability and ROS production compared with cells that did not express this p53 isoform (51).

## p53 and the ER and Mitochondria-Associated ER Membranes

Another important subcellular localization of p53 is the ER, where it plays a critical role in the modulation of apoptosis and autophagy.

p53 regulates autophagy in a dual fashion: the pool of nuclear p53 stimulates autophagy in a transcription-dependent fashion (52, 53) and the pool of cytoplasmic p53 protein represses autophagy in a transcription-independent manner (54).

Suppression of autophagy is mediated by cytoplasmic, not nuclear p53. Indeed, Tasdemir at al. observed that p53 KO cells display higher autophagy levels compared to their wt counterpart. This effect was reverted by overexpression of wt p53, p53 with impaired nuclear localization sequence, and ER-targeted p53 (p53ER). A p53 form that was unable to exit the nucleus [using a disrupted nuclear export signal (NES)] failed to inhibit autophagy and the p53 inhibitor pifitrin α was able to induce autophagy in wt cells or nucleus deprived cells (54). Interestingly, the R175H mutation, which is known to inhibit the nuclear and cytoplasmic effects of p53 (34, 55), prevented inhibition of autophagy (54).

These results indicate that p53 inhibits autophagy through a transcription-independent effect exerted from a cytoplasmic localization. In fact, cytoplasmic p53 inhibits the AMP-dependent kinase, a positive regulator of autophagy, and activates mammalian target of rapamycin (mTOR), a negative regulator of autophagy. At present, the exact molecular pathway by which autophagy-inducing stimuli, such as ER stress, cause the cytoplasmic translocation and subsequent degradation of p53 remain unknown. It is known that its sequential phosphorylation of p53 on Ser 315 and Ser 376, the nuclear export, the ubiquitination by HDM2 and its proteasome-mediated degradation is required (56, 57). Inhibiting HDM2 or the proteasome prevents degradation of p53 induced by various autophagy triggers and inhibits autophagy.

In recent years, many other tumor suppressor proteins, such as PML and PTEN, have been demonstrated to localize to the ER and MAMs, where they regulate the ER–mitochondria Ca^2+^ flux and apoptosis (58, 59). p53 localization at the ER has been previously suggested (60), but this specific ER-localization was unable to regulate cell death induced by calcium-independent apoptotic stimuli.

We demonstrated that in the cytoplasm, wt p53 localizes at the ER and MAMs to modulate Ca^2+^-mediated apoptosis in a transcription-independent manner (61). This non-nuclear fraction of p53 is able to modulate Ca^2+^ homeostasis in response to both physiological and pathological stimulation; in fact, activation and accumulation of p53 at the ER/MAMs render cells more prone to death, and the absence of p53 leads to lower steady-state levels of reticular Ca^2+^, reduced Ca^2+^ mobilization, and mitochondrial accumulation evoked by agonist stimulation (ATP) or after the oxidative apoptotic inducer H_2_O_2_ (Figure 1). Importantly, to exclude the possibility that these effects are independent from the transcriptional activity of p53, different experimental strategies were used: a pharmacological inhibition of the transcriptional arm of p53 through the use of RNA polymerase II inhibitor α-amanitin (62), alone or in combination with pifitrin α, and the overexpression of p53 mutants lacking nuclear localization signal (p53 ΔNLS or ER p53).

Thus, p53 controls mitochondrial Ca^2+^ homeostasis and, in turn, apoptotic sensitivity from the ER/MAMs compartments.

Various naturally occurring p53 mutants, such as p53 R175H and p53 R273H, are unable to restore ER Ca^2+^ homeostasis when overexpressed in p53 KO cells, while the wt p53 efficiently does so. Accordingly, overexpression of wt p53, but not p53 R175H and p53 R273H, increased the sensitivity of p53 KO cells to oxidative stress back to the levels of their p53^+/+^ counterparts, although there were no differences in the expression of apoptotic genes in cells expressing mutant p53. Moreover, cells harboring the p53 R273H mutation, such as MDA-MB 468 cells, did not display any significant alterations in ER Ca^2+^ levels when p53 was stabilized by adriamycin. Next, it was demonstrated that the Sarco/ER Ca^2+^-ATPase Pump (SERCA), a pump responsible for maintaining high Ca^2+^ levels in the ER lumen (63), selectively binds wt p53 on the C-terminal portion, modulating its oxidation status; however, this domain alone is not sufficient to modulate Ca^2+^ homeostasis and apoptosis, indicating that this function requires the entire protein. Interestingly, the naturally occurring p53 mutants R175H and R273H were unable to bind SERCA and its oxidation was unchanged.

Taken together, all of these data suggest that Ca^2+^-mediated apoptosis is a transcription-independent pathway regulated by p53 at ER/MAMs, through which p53 exerts its potent proapoptotic role in response to anticancer treatments.

To elucidate the relevance of these findings *in vivo*, we investigated the involvement of p53 in the control of intracellular Ca^2+^ signals and apoptosis in a 3D tumor mass in living mice (64). The use of a “skinfold chamber” installed on the back of athymic mice allowed the monitoring of tumor formation and, through a single-photon fluorescence microscope, the investigation of Ca^2+^ dynamics inside the tumor.

When detectable, the mass was stained with Fura-2, a Ca^2+^-sensitive dye, and aluminum phtalocyanine chloride, a light-activated agent used in cancer photodynamic therapy (PDT). PDT accumulates in intracellular organelles, including ER and mitochondria, and after photo stimulation, it promotes Ca^2+^-dependent apoptotic pathways. Through this technique, we demonstrated that p53 is able to modulate the Ca^2+^ response and that this is associated with reduced responsiveness to apoptotic stimulation. Together, these results reveal a new mechanism by which p53 exerts its potent proapoptotic function in response to anticancer treatments.

Importantly, p53 can not only regulate cell survival through its activity at ER/MAMs sites but also alter ER functions that can control both localization and apoptotic activity of p53. It has been shown that ER stress inhibits p53-mediated apoptosis, modulating its localization and function (57). In fact, ER stress induces the cytoplasmic localization and enhances the destabilization of p53 due to phosphorylation at serine 315 and serine 376, which is mediated by glycogen synthase kinase-3-β (GSK3-β). As a result of the increased cytoplasmic localization, ER stress prevents p53 stabilization and p53-mediated apoptosis in response to DNA damage (57). Furthermore, it has been demonstrated that induction of the cytoplasmic translocation and degradation of p53 by ER stress is mediated by Hdm2 (56). This could have possible important implications for treatment of tumors with dysfunctional ER, aiming at p53 stabilization through the inhibition of the p53–Hdm2–GSK3-β pathway. Overall, these findings suggest that the cross talk of p53 with the dynamic ER plays a pivotal role in the regulation of cell survival and provides important evidence on how the specific targeting of the ER by tumor suppressors could counteract tumor progression.

## Future Perspectives

Despite the long-time knowledge on p53 involvement in tumorigenesis, its translation to clinical field has yet to be concluded. The studies summarized above clearly confirm that transcription-independent activities of p53 play an important role in the ability of the protein to activate several pathways in many circumstances. Nevertheless, many efforts still need to dissect the intricate signaling network that coordinates and couples the transcriptional and non-transcriptional proapoptotic activities of p53. In this way, there are still various mutants to be characterized, and how naturally occurring mutations affect p53 structure and function also remains elusive, as does the role of loss of wt and gain-of-function amino acid substitutions. Moreover, p53 extra-transcription activity studies using p53 mutants’ overexpression should consider possible transcription activity alteration. For instance, high concentrations of p53 are demonstrated to inhibited p53-activated transcription by squelching (65, 66). Further studies should clarify how transcription and extra-transcription effects could cooperate.

This recognition and classification that not all p53 mutants are equivalent is important not merely as a conceptual distinction but may also have practical implications.

## Author Contributions

All authors listed, have made substantial, direct and intellectual contribution to the work, and approved it for publication.

## Conflict of Interest Statement

The authors declare that the research was conducted in the absence of any commercial or financial relationships that could be construed as a potential conflict of interest.
